# Evaluation of a Lyophilized CRISPR-Cas12 Assay for a Sensitive, Specific, and Rapid Detection of SARS-CoV-2

**DOI:** 10.3390/v13030420

**Published:** 2021-03-05

**Authors:** Lucía Ana Curti, Ivana Primost, Sofia Valla, Daiana Ibañez Alegre, Cecilia Olguin Perglione, Guillermo Daniel Repizo, Julia Lara, Ivana Parcerisa, Antonela Palacios, María Eugenia Llases, Adriana Rinflerch, Melanie Barrios, Federico Pereyra Bonnet, Carla Alejandra Gimenez, Débora Natalia Marcone

**Affiliations:** 1 CASPR Biotech, San Francisco, CA 94103, USA; lucia@caspr.bio (L.A.C.); guillermo@caspr.bio (G.D.R.); julia.lara@caspr.bio (J.L.); iva.parce@caspr.bio (I.P.); anto.palacios@caspr.bio (A.P.); maru.llases@caspr.bio (M.E.L.); federico@caspr.bio (F.P.B.); 2 Genetics and Molecular Biology Laboratory, Hospital Municipal de Trauma y Emergencias Dr. Federico Abete, Malvinas Argentinas, Buenos Aires 1615, Argentina; ivanaprimost@gmail.com; 3 Centro de Investigaciones Básicas y Aplicadas (CIBA), Centro de Investigaciones y Transferencia del Noroeste de la Provincia de Buenos Aires (CITNOBA), Universidad del Noroeste de la Provincia de Buenos Aires (UNNOBA), Junín, Buenos Aires 6000, Argentina; sofiaavalla@gmail.com; 4 CONICET-Consejo Nacional de Investigaciones Científicas y Técnicas, Buenos Aires 1425, Argentina; daianamacarenaib@gmail.com (D.I.A.); ceciolpergli@gmail.com (C.O.P.); rinflerch@gmail.com (A.R.); 5 Laboratorio Grupo de Investigación en Genética Aplicada (GIGA, FCEQyN), Instituto de Biología Subtropical, Universidad Nacional de Misiones (UNM–CONICET), Posadas 3304, Argentina; 6 Instituto de Virología, Instituto Nacional de Tecnología Agropecuaria (INTA), Buenos Aires 1686, Argentina; 7 Instituto de Producción Agropecuaria (INPA), Universidad de Buenos Aires, Buenos Aires 1053, Argentina; melaniebarrios@outlook.com; 8 Departamento de Microbiología, Inmunología, Biotecnología y Genética, Cátedra de Virología, Facultad de Farmacia y Bioquímica, Universidad de Buenos Aires, Buenos Aires 1113, Argentina

**Keywords:** CRISPR, SARS-CoV-2, COVID-19, diagnosis, fluorescence detection, lysis buffer

## Abstract

We evaluated a lyophilized CRISPR-Cas12 assay for SARS-CoV-2 detection (Lyo-CRISPR SARS-CoV-2 kit) based on reverse transcription, isothermal amplification, and CRISPR-Cas12 reaction. From a total of 210 RNA samples extracted from nasopharyngeal swabs using spin columns, the Lyo-CRISPR SARS-CoV-2 kit detected 105/105 (100%; 95% confidence interval (CI): 96.55–100) positive samples and 104/105 (99.05%; 95% CI: 94.81–99.97) negative samples that were previously tested using commercial RT-qPCR. The estimated overall Kappa index was 0.991, reflecting an almost perfect concordance level between the two diagnostic tests. An initial validation test was also performed on 30 nasopharyngeal samples collected in lysis buffer, in which the Lyo-CRISPR SARS-CoV-2 kit detected 20/21 (95.24%; 95% CI: 76.18–99.88) positive samples and 9/9 (100%; 95% CI: 66.37–100) negative samples. The estimated Kappa index was 0.923, indicating a strong concordance between the test procedures. The Lyo-CRISPR SARS-CoV-2 kit was suitable for detecting a wide range of RT-qPCR-positive samples (cycle threshold range: 11.45–36.90) and dilutions of heat-inactivated virus (range: 2.5–100 copies/µL); no cross-reaction was observed with the other respiratory pathogens tested. We demonstrated that the performance of the Lyo-CRISPR SARS-CoV-2 kit was similar to that of commercial RT-qPCR, as the former was highly sensitive and specific, timesaving (1.5 h), inexpensive, and did not require sophisticated equipment. The use of this kit would reduce the time taken for diagnosis and facilitate molecular diagnosis in low-resource laboratories.

## 1. Introduction

Since December 2019, severe acute respiratory syndrome coronavirus 2 (SARS-CoV-2) has spread worldwide causing an outbreak of a respiratory disease, now known as coronavirus disease (COVID-19) [1]. Approximately 107.8 million COVID-19 cases and 2.3 million associated deaths have been reported until 13 February 2021 [2]. The need for a rapid, sensitive, and specific diagnostic tool, in addition to the requirement of an adequate supply of reagents, has led researchers to explore different methods for SARS-CoV-2 detection other than the gold standard method of RT-qPCR [3], such as isothermal amplification techniques combined with colorimetric detection [4,5,6]. Although RT-qPCR tests can be performed without sophisticated equipment and do not require the expertise of well-trained personnel, this technique is prone to detecting unspecific signals, which may lead to false-positive results. However, the combination of RNA-guided clustered regularly interspaced short palindromic repeats (CRISPR) with Cas effectors improves the accuracy of results due to the collateral cleavage of a reporter molecule in the presence of a target, showing a performance comparable to that of RT-qPCR for viral and bacterial detection [7,8], including SARS-CoV-2 (Table S1) [9,10,11,12,13,14].

To evaluate the Lyo-CRISPR SARS-CoV-2 kit (CASPR Biotech, San Francisco, CA, USA) based on reverse transcription, isothermal amplification and CRISPR-Cas12 detection, we tested respiratory samples from patients with COVID-19 symptoms. The kit is authorized by the Argentine Administración Nacional de Medicamentos, Alimentos y Tecnología Médica to be distributed in Argentina. The analytical sensitivity of the kit for testing the extracted RNA samples was 7.5 copies/µL (within the range of commercial RT-qPCR [100–1000 copies/mL]) [15]; clinical sensitivity and specificity were 99.03% and 99.04%, respectively, and no cross-reactivity was observed.

## 2. Materials and Methods

### 2.1. Study Design

A cross-sectional study was conducted from April 1 to June 30, 2020, in Buenos Aires, Argentina. A total of 210 respiratory samples previously tested by RT-qPCR using a commercial kit for SARS-CoV-2 detection (GeneFinder COVID-19 Plus [OSANG Healthcare Co, Anyang-si, Korea]) were used. The samples were randomly selected from 105 positive (cycle threshold [Ct] range: 8–37) and 105 negative patients with COVID-19 symptoms hospitalized at Dr. Abete Municipal Hospital. Nasopharyngeal swabs are the specimen of choice because they have the highest diagnostic performance for the detection of SARS-CoV-2 and other respiratory viruses. Moreover, the collection of nasopharyngeal swabs is recommended by the World Health Organization and US Centers for Disease Control and Prevention. The protocol was reviewed and approved by the institutional review board of Dr. Abete Municipal Hospital. No consent was required from the subjects because all data were analyzed anonymously.

### 2.2. Lyo-CRISPR Assay

**Figure 1:**
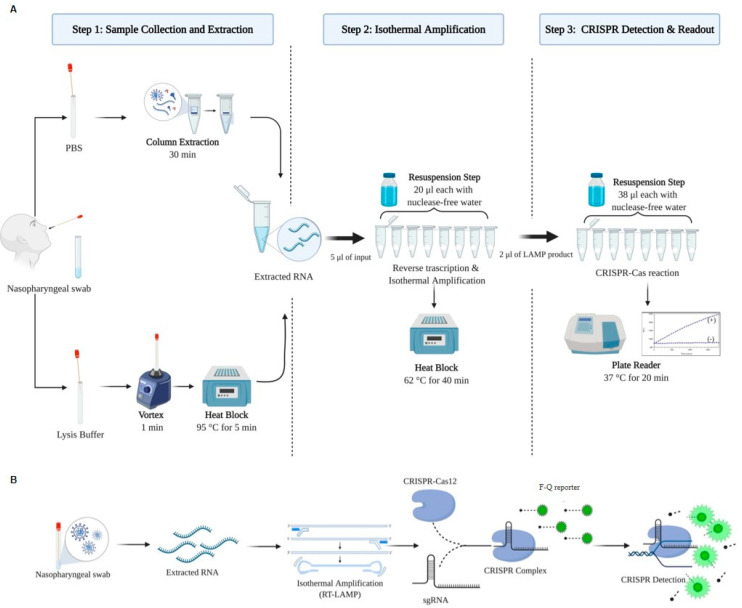
Lyo-CRISPR SARS-CoV-2 assay. (**A**) Processing scheme, including the reagents and equipment required for performing the following three steps: RNA extraction using spin columns or by treating the samples with lysis buffer and heat, to be used as input in the reverse transcription (RT) and isothermal amplification reactions and CRISPR-Cas12 detection for N gene by fluorescence. Resuspension with nuclease-free water is required to hydrate the lyophilized beads containing all components for isothermal amplification and CRISPR detection. (**B**) Workflow scheme showing the entire process at molecular level. PBS: Phosphate Buffered Saline buffer; min: minutes; Lyo: lyophilized; SARS-CoV-2: severe acute respiratory syndrome coronavirus 2; CRISPR: clustered regularly interspaced short palindromic repeats; sgRNA: single guide RNA; F-Q reporter: fluorophore-quencher reporter.

Viral RNA was extracted using spin columns with QIAamp Viral RNA Mini Kit (Qiagen, Hilden, Germany), according to the manufacturer’s instructions. Although the Lyo-CRISPR SARS-CoV-2 kit is authorized for use on extracted RNA, a first assay was performed using samples collected in lysis buffer. For this purpose, 30 nasopharyngeal swabs (collected from 21 positive and 9 negative hospitalized patients with COVID-19 symptoms) previously analyzed using RT-qPCR were collected in 500 µL of lysis buffer (QuickExtract DNA; Lucigen, Middleton, WI, USA) (Figure 1A,B). The samples in lysis buffer were mixed by vortexing for 15 s and heated at 95 °C for 5 min to release the viral RNA. RNA extracted either using spin columns or lysis buffer and heating was tested using the Lyo-CRISPR SARS-CoV-2 kit, which detects a target region in the nucleocapsid (N) gene. The results were compared with those of GeneFinder COVID-19 Plus, which detects the RNA-dependent RNA polymerase (RdRp), envelope (E), and N genes.

The Lyo-CRISPR SARS-CoV-2 assay was initiated by adding 20 µL of PCR-grade water to an 8-strip tube with lyophilized beads containing all optimized components to perform the isothermal amplification step. The reaction was carried out at 62 °C for 40 min after adding 5 µL of the extracted RNA. For the CRISPR-Cas12 detection step, 38 µL of PCR-grade water was added to a second 8-strip tube with lyophilized beads containing all optimized components, and a 2 µL aliquot of the amplified product was incubated at 37 °C for 20 min. Fluorescence was measured (λ_ex_ = 485 nm; λ_em_ = 535 nm) using an Infinite 200 plate reader (Tecan Trading, Switzerland). A positive result was considered if the fluorescence ratio (R) at 20 min showed a minimum 2.5-fold increase between the sample and non-template control reaction (R = Intensity fluorescence (IF)_t20_ sample/IF_t20_ non-template control). The result was considered negative if R was lower than 2.5-fold.

For each respiratory sample, two reactions were performed in parallel. One in 8-strip tubes called “N-gene,” which contain specific primers and sgRNA targeting the viral N gene, and another reaction in different 8-strip tubes called “RNAseP,” which include specific primers and sgRNA for the detection of the RNAseP gene as an internal control.

### 2.3. Analytical Sensitivity

Analytical sensitivity was evaluated using a standard curve for 5-fold dilutions of heat-inactivated SARS-CoV-2 control samples (ATCC^®^ VR-1986HK^TM^ Manassas, VA, USA). Contrived samples were prepared as follows: oropharyngeal swabs obtained from healthy donors were collected in 500 µL of phosphate-buffered saline and vortexed for 2 min to obtain a homogeneous human respiratory matrix; heat-inactivated virus at concentrations of 100, 10, 7.5, 5, and 2.5 copies/µL was spiked into this matrix. RNA was extracted from each dilution using commercial spin columns (QIAamp Viral RNA Mini Kit), and the eluted RNA was used as input for the Lyo-CRISPR SARS-CoV-2 reaction. Three replicates were performed for each dilution. Three controls were added to verify all steps of the whole reaction: a non-template control that uses nuclease-free water as the input of reaction, an amplification control tube that includes water as the input and amplification beads without the CRISPR reaction, and a CRISPR control tube that only includes water as the input and CRISPR beads, but without the amplification reaction.

### 2.4. Specificity

For evaluating specificity, a total of 21 commercial respiratory pathogen controls were tested using the Lyo-CRISPR SARS-CoV-2 kit. Commercial controls were selected to include other human coronaviruses, such as CoV-229E, CoV-OC43, CoV-HKU1, CoV-NL63, SARS-CoV-1, MERS-CoV, and other pathogens of the respiratory tract, including respiratory syncytial virus, influenza A and B, rhinovirus, *Mycobacterium tuberculosis, Streptococcus pyogenes, Streptococcus pneumoniae, Chlamydia pneumoniae, Bordetella pertussis, Haemophilus influenzae, Legionella pneumophila, Streptococcus salivarius, Candida albicans, Pseudomonas aeruginosa,* and *Staphylococcus epidermis.* Each commercial respiratory control was spiked into the human respiratory matrix, following which RNA was extracted with commercial columns, and the eluate was tested in triplicate.

### 2.5. Statistical Analysis

Concordance measures (percent positive, negative, and overall agreement) and their two-sided 95% confidence intervals (95% CI) between the results of respiratory sample tests using both the diagnostic methods were calculated and complemented with the Cohen’s Kappa test. All analyses were performed using STATA 12 software (StataCorp, College Station, TX, USA).

## 3. Results

### 3.1. Performance of the Lyo-CRISPR SARS-CoV-2 Kit in the Extracted RNA Samples

**Figure 2:**
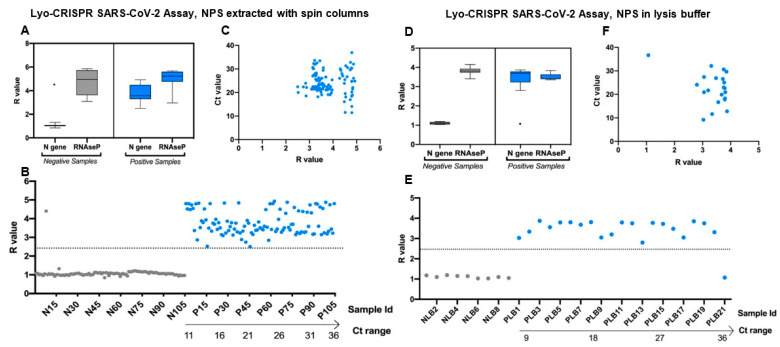
Lyo-CRISPR SARS-CoV-2 kit results of 210 RNA samples extracted from nasopharyngeal swabs (NPS) using spin columns (**A**–**C**) and 30 RNA samples extracted from nasopharyngeal swabs with direct lysis (**D**–**F**). (**A**,**D**) Lyo-CRISPR SARS-CoV-2 kit results for N gene and internal control (RNAseP) in RT-qPCR-negative (grey) and -positive (blue) samples. Median, interquartile range, and range of the fluorescence ratio (R = intensity fluorescence (IF)_t20_ sample/IF_t20_ non-template control) values measured at 20 min are shown. A positive result was considered if R ≥ 2.5 or negative if R ˂ 2.5. (**B**,**E**) R values obtained for each RT-qPCR-negative (grey) and -positive (blue) sample tested. Each dot represents one sample and the dashed line represents the cutoff of the Lyo-CRISPR test to classify them. Positive samples were sorted by Ct values determined using the GeneFinder RT-qPCR kit (N gene). (**C**,**F**) R values obtained for each RT-qPCR-positive sample tested. Each dot represents one sample. N: nucleocapsid; Id: identification; Ct: Cycle threshold, R: fluorescence ratio. Figures were designed with GraphPad Prism V8 software (GraphPad, San Diego, California, USA) and BioRender.com.

Of the 210 RNA samples extracted, the Lyo-CRISPR SARS-CoV-2 kit detected 105/105 (100%) positive samples and 104/105 (99.05%) negative samples previously tested by RT-qPCR (Figure 2A–C). All results were validated by a positive result for the RNAseP reaction (Tables S2 and S3). The time required to perform the assay, including the extraction step was 1 h 40 min. The estimated negative, positive, and overall agreement was >99% (Table 1). The overall kappa index was 0.991, reflecting an almost perfect concordance level between the two diagnostic tests. Regarding positive samples, the performance of the Lyo-CRISPR SARS-CoV-2 kit was suitable for detecting a wide range of RT-qPCR Ct values (N gene Ct range: 11.45–36.90), which was satisfactory for respiratory samples with high, medium, or low viral loads.

### 3.2. Performance of the Lyo-CRISPR SARS-CoV-2 Kit in Samples Collected in Lysis Buffer

Of the 30 respiratory samples collected in lysis buffer, the Lyo-CRISPR SARS-CoV-2 kit detected 20/21 (95.24%) RT-qPCR-positive samples and 9/9 (100%) RT-qPCR negative samples (Figure 2D–F). The time required to perform the whole assay for testing simultaneously all samples was 70 min. The results of both diagnostic tests corresponded in 95.24% of positive samples; the overall agreement reached up to 96.67%, and the Kappa index was 0.923, indicating a strong agreement between the two test procedures (Table 1). The Lyo-CRISPR assay was suitable for detecting positive samples with a Ct range (N gene) of 9.17–32.08. (Table S4). The only positive sample that the Lyo-CRISPR assay failed to detect had a Ct value (N gene) of 36.65, showing that a low viral load could be the reason for this negative result.

### 3.3. Analytical Sensitivity

The Lyo-CRISPR SARS-CoV-2 kit was suitable for detecting all dilutions of heat-inactivated viruses performed within the range of 2.5–100 copies/µL (Table 2, Figure S1). In our tests, three replicates were obtained for all dilutions except one (10 copies/µL), for which two replicates (out of three) were obtained. However, the three replicates of the lowest dilutions were positive. The three negative controls were negative for the N gene and were validated by a positive result for the RNAseP reaction.

### 3.4. Specificity

All respiratory pathogens controls, including other human coronaviruses and respiratory viruses, bacteria, chlamydia, and mycoplasma tested were negative for the N gene reaction and were validated with the RNAseP reaction (Table 3).

## 4. Discussion

The performance of the Lyo-CRISPR SARS-CoV-2 kit was satisfactory for both respiratory samples tested, i.e., those extracted with spin columns and those collected in lysis buffer. Although the assay was not developed or authorized for use in samples collected in lysis buffer, its performance was satisfactory, suggesting that with adequate optimization and a new regulatory clearance to market, this assay could be used for SARS-CoV-2 detection, saving considerable time and costs by avoiding the RNA isolation step and offering a marked advantage over RT-qPCR. One possible adjustment would be to increase the input volume when using a lysis buffer with the aim of increasing sensitivity.

In addition, the Lyo-CRISPR SARS-CoV-2 kit proved to be sensitive to all dilutions of the SARS-CoV-2 control tested, ranging from 2.5–100 copies/µL. The limit of detection of the Lyo-CRISPR SARS-CoV-2 kit was 7.5 copies/µL, which was similar to that of other assays based on CRISPR-Cas technology for SARS-CoV-2 detection, such as 6.75 copies/µL reported by SHERLOCK^®^ and 20 copies/µL reported by DETECTER^®^ kits. No cross-reaction was observed when 6 commercial human coronaviruses and 15 respiratory pathogens, including viruses and bacteria, were tested with the Lyo-CRISPR SARS-CoV-2.

Assay time ranged from 1 h 40 min if the sample was extracted with spin columns to 70 min when the samples collected in lysis buffer were used. The assay times for both were within the range of most commercial RT-qPCR assays. The main advantage of this method was that it did not require the use of expensive equipment and highly qualified personnel and was stable at room temperature (15–30 °C), due to the lyophilized format of reagents, which make this kit more stable and easier to transport and store. Furthermore, its associated cost (US$ 12/reaction) was lower than that of commercial RT-qPCR, which was further reduced if the sample was collected in lysis buffer.

The Lyo-CRISPR SARS-CoV-2 kit has a few limitations. It targets only one region in the SARS-CoV-2 genome, and this could affect the robustness of this assay due to the continued evolution of viral strains. One possible approach would be to incorporate another target region into its updated version. However, our results showed that the assay was adequate for detecting positive samples obtained over a period of three months. Another limitation is that the tube containing amplified products must be opened to perform CRISPR detection, which could be a potential source of contamination. However, during the course of the current study, no contamination occurred due to good laboratory practices, use of disposable materials, unidirectional workflow, separate areas with dedicated equipment and supplies for nucleic acid extraction, RT-isothermal amplification step, and CRISPR detection. After each reaction, laboratory waste was removed, and work surfaces, pipettes, and centrifuges were cleaned and decontaminated with cleaning products and equipment, such as 20% bleach, RNase AWAY^®^, and UV light.

## 5. Conclusions

Since the concordance between the two diagnostic tests was almost perfect for both samples tested, our results showed that the Lyo-CRISPR SARS-CoV-2 kit was a rapid, inexpensive, sensitive, and specific method for SARS-CoV-2 detection, and it could serve as an alternative to RT-qPCR.

## Figures and Tables

**Table 1:** Agreement between GeneFinder RT-qPCR and Lyo-CRISPR SARS-CoV-2 kit results for testing samples extracted with spin column (*n* = 210) and samples in lysis buffer (*n* = 30) from hospitalized patients.

Respiratory Samples	GeneFinder RT-qPCR			Lyo-CRISPR SARS-CoV-2		Agreement		Overall Agreement		Cohen Kappa	
	E Gene	N Gene	RdRp Gene	Negative	Positive						
RT-qPCR result	Ct mean (range)	Ct mean (range)	Ct mean (range)	*n*	*n*	% (95%CI)		% (95%CI)		k (95%CI)	
RNA extracted											
Negative (*n* = 105)	-	-	-	104	1	99.05	(94.81–99.97)	99.52	(97.38–99.99)	0.991	(0.972–1)
Positive (*n* = 105)	25.65 (10.83–40.02)	24.19 (11.45–36.90)	24.03 (11.36–34.27)	0	105	100	(96.55–100)				
Lysis Buffer											
Negative (*n* = 9)	-	-	-	9	0	100	(66.37–100)	96.67	(82.78–99.92)	0.923	(0.775–1)
Positive (*n* = 21)	21.33 (9.10–35.60)	22.58 (9.17–36.65)	19.57 (8.30–34.32)	1	20	95.24	(76.18–99.88)				

Lyo: lyophilized; SARS-CoV-2: severe acute respiratory syndrome coronavirus 2; CRISPR: clustered regularly interspaced short palindromic repeats; 95%CI: 95% confidence interval; Ct: cycle threshold.

**Table 2:** Analytical sensitivity evaluation of Lyo-CRISPR SARS-CoV-2 kit.

	Lyo-CRISPR SARS-CoV-2			
	N Gene	N Gene Result	RNAseP	RNAseP Result
SARS-CoV-2 Control				
100 copies/µL	3/3	Positive	3/3	Valid
10 copies/µL	2/3	Positive	3/3	Valid
7.5 copies/µL	3/3	Positive	3/3	Valid
5 copies/µL	3/3	Positive	3/3	Valid
2.5 copies/µL	3/3	Positive	3/3	Valid
Non-template Control	0/3	Negative	3/3	Valid
Amplification Control	0/3	Negative	3/3	Valid
CRISPR Control	0/3	Negative	3/3	Valid

SARS-CoV-2: severe acute respiratory syndrome coronavirus 2; CRISPR: clustered regularly interspaced short palindromic repeats.

**Table 3:** Cross-reaction evaluation of Lyo-CRISPR SARS-CoV-2 kit with other respiratory pathogens.

Pathogens	Source Cat. Number	Tested Concentration	Lyo-CRISPR SARS-CoV-2			
			N Gene	N Gene Result	RNAseP	RNAseP Result
Human coronavirus 229E	0810229CFHI	10^5^ TCID50/mL	0/3	Negative	3/3	Valid
Human coronavirus OC43	0810024CFHI	10^5^ TCID50/mL	0/3	Negative	3/3	Valid
Human coronavirus HKU1	ATCC^®^ VR-3262SD	10^5^ copies/mL	0/3	Negative	3/3	Valid
Human coronavirus NL63	ATCC^®^ 3263SD	10^5^ copies/mL	0/3	Negative	3/3	Valid
SARS-CoV	NATSARS-ST	10^5^ TCID50/mL	0/3	Negative	3/3	Valid
MERS-CoV	NR-45843	10^5^ copies/mL	0/3	Negative	3/3	Valid
Respiratory syncytial virus	ATCC^®^ VR-1580DQ	10^5^ copies/mL	0/3	Negative	3/3	Valid
Influenza A H1N1	ATCC^®^ VR-95DQ	10^5^ copies/mL	0/3	Negative	3/3	Valid
Influenza B (Yamagata Lineage)	ATCC^®^ VR-1885DQ	10^5^ copies/mL	0/3	Negative	3/3	Valid
Rhinovirus	NR-51453	10^5^ copies/mL	0/3	Negative	3/3	Valid
*Mycobacterium tuberculosis*	NR-14867	10^6^ copies/mL	0/3	Negative	3/3	Valid
*Streptococcus pyogenes*	ATCC^®^ 12344D-5	10^6^ copies/mL	0/3	Negative	3/3	Valid
*Streptococcus pneumoniae*	ATCC^®^ 700669D-5	10^6^ copies/mL	0/3	Negative	3/3	Valid
*Chlamydia pneumoniae*	ATCC^®^ VR-1360D	10^6^ copies/mL	0/3	Negative	3/3	Valid
*Bordetella pertussis*	ATCC^®^ 9797D-5	10^6^ copies/mL	0/3	Negative	3/3	Valid
*Haemophilus influenzae*	ATCC^®^ 51907D-5	10^5^ copies/mL	0/3	Negative	3/3	Valid
*Legionella pneumophila*	ATCC^®^ 33152D-5	10^6^ copies/mL	0/3	Negative	3/3	Valid
*Streptococcus salivarius*	HM-121D	10^6^ copies/mL	0/3	Negative	3/3	Valid
*Candida albicans*	ATCC^®^ 10231D-5	10^6^ copies/mL	0/3	Negative	3/3	Valid
*Pseudomonas aeruginosa*	ATCC^®^ 27853D-5	10^6^ copies/mL	0/3	Negative	3/3	Valid
*Staphylococcus epidermis*	ATCC^®^ 12228D-5	10^6^ copies/mL	0/3	Negative	3/3	Valid

Lyo: lyophilized; SARS-CoV-2: severe acute respiratory syndrome coronavirus 2; CRISPR: clustered regularly interspaced short palindromic repeats; ATCC: American Type Culture Collection; TCID50: Tissue Culture Infectious Dose 50%.

## Data Availability

Not applicable.

## Supplementary Materials

The following are available online at https://www.mdpi.com/1999-4915/13/3/420/s1, Figure S1: Analytical sensitivity evaluation of Lyo-CRISPR SARS-CoV-2 kit, Table S1: Comparison of CRISPR-Cas based methods for SARS-CoV-2 detection in respiratory samples, Table S2: Lyo-CRISPR SARS-CoV-2 kit results of 105 negative RT-qPCR samples; Table S3: Lyo-CRISPR SARS-CoV-2 kit results of 105 positive RT-qPCR samples; Table S4: Lyo-CRISPR SARS-CoV-2 kit results of 30 samples collected in lysis buffer.
