# Supplementary material for: Evaluation of a Lyophilized CRISPR-Cas12 Assay for a Sensitive, Specific, and Rapid Detection of SARS-CoV-2
Source: Viruses. 2021 Mar 5;13(3):420. doi: 10.3390/v13030420 (PMC7998296; doi:10.3390/v13030420)
Supplement: Supplementary file 1 [file viruses-13-00420-s001.zip › viruses-1106900-supplementary/SuppData/Figure S1.docx]

**Figure S1**. Analytical sensitivity evaluation of Lyo-CRISPR SARS-CoV-2 kit


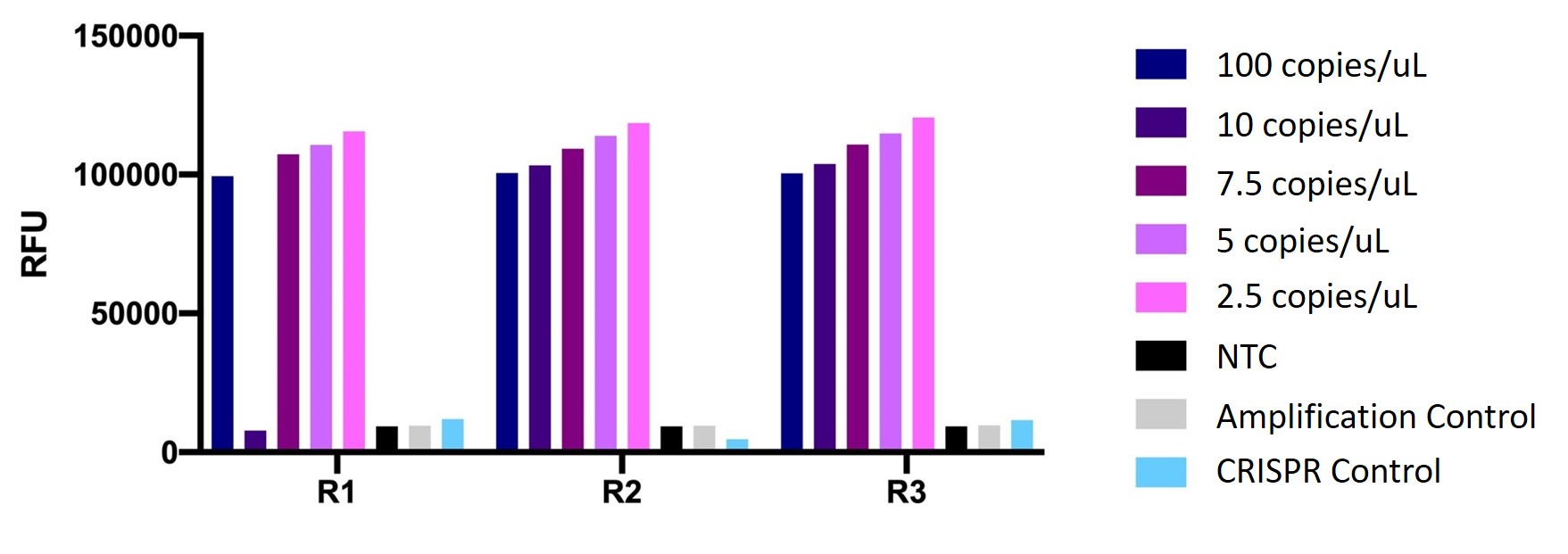


Figure S1. Legend

**Analytical sensitivity evaluation of Lyo-CRISPR SARS-CoV-2 kit.**

Fluorescence signal of a standard curve that included 5 dilutions of heat inactivated SARS-CoV-2 control and 3 negative controls tested with the Lyo-CRISPR SARS-CoV-2 kit, in triplicate.

RFU: reference fluorescence unit; R1-3: replicate 1 – 3.
