# Supplementary material for: Evaluation of a Lyophilized CRISPR-Cas12 Assay for a Sensitive, Specific, and Rapid Detection of SARS-CoV-2
Source: Viruses. 2021 Mar 5;13(3):420. doi: 10.3390/v13030420 (PMC7998296; doi:10.3390/v13030420)
Supplement: Supplementary file 1 [file viruses-13-00420-s001.zip › viruses-1106900-supplementary/SuppData/Table S1.docx]

**Table S1**. Comparison of CRISPR-Cas based methods for SARS-CoV-2 detection in respiratory samples

|  | **CASPR Lyo-CRISPR SARS-CoV-2 Kit** | **SHERLOCK® CRISPR SARS-CoV-2 Kit** | **SARS-CoV-2 DETECTR® Reagent Kit** |
| --- | --- | --- | --- |
| Sample Collection | Nasopharyngeal/oropharyngeal swabs specimens | Upper respiratory specimens | Upper respiratory specimens |
| Sample Type | Extracted RNA | Extracted RNA | Extracted RNA |
| Technology | RT-Isothermal Amplification + CRISPR-Cas12 detection | Isothermal Amplification + CRISPR-Cas13 detection | RT-Isothermal Amplification + CRISPR-Cas12 detection |
| Gene target/s | N / RNAse P | ORF1ab / N / RNAseP | N / RNAse P |
| Limit of Detection | 7.5 copies/ ul | 6.75 copies /ul VTM | 20 copies/ ul |
| Shipping Condition | Room temperature | Dry ice | Dry ice |
| Storage Condition | 2-8 ºC | -20 ºC | -20 ºC |
| Fluorescents measurement | Fluorescence Plate Reader | Fluorescence Plate Reader | Fluorescence Plate Reader |
| Format | Lyophilized | Liquid | Liquid |

Lyo: lyophilized; SARS-CoV-2: severe acute respiratory syndrome coronavirus 2; CRISPR: clustered regularly interspaced short palindromic repeats
