# Supplementary material for: Evaluation of a Lyophilized CRISPR-Cas12 Assay for a Sensitive, Specific, and Rapid Detection of SARS-CoV-2
Source: Viruses. 2021 Mar 5;13(3):420. doi: 10.3390/v13030420 (PMC7998296; doi:10.3390/v13030420)
Supplement: Supplementary file 1 [file viruses-13-00420-s001.zip › viruses-1106900-supplementary/SuppData/Table S2.docx]

**Table S2.** Lyo-CRISPR results of 105 negative RT-qPCR samples.

| Sample Id | GeneFinder RT-qPCR |  | Lyo-CRISPR SARS-CoV-2 | | | | |
| --- | --- | --- | --- | --- | --- | --- | --- |
|  |  |  | N gene | |  | RNAseP | |
|  | Result |  | R (IF_t20_/IF_t20NTC_) | Result |  | R (IF_t20_/IF_t20NTC_) | Result |
| N1 | Negative |  | 1.07 | Negative |  | 4.88 | Valid |
| N2 | Negative |  | 1.07 | Negative |  | 4.90 | Valid |
| N3 | Negative |  | 1.00 | Negative |  | 4.81 | Valid |
| N4 | Negative |  | 1.02 | Negative |  | 4.91 | Valid |
| N5 | Negative |  | 0.99 | Negative |  | 4.90 | Valid |
| N6 | Negative |  | 0.96 | Negative |  | 4.95 | Valid |
| N7 | Negative |  | 1.05 | Negative |  | 5.03 | Valid |
| N8 | Negative |  | 4.40 | Positive |  | 4.98 | Valid |
| N9 | Negative |  | 1.00 | Negative |  | 4.99 | Valid |
| N10 | Negative |  | 1.05 | Negative |  | 3.86 | Valid |
| N11 | Negative |  | 1.02 | Negative |  | 4.99 | Valid |
| N12 | Negative |  | 1.04 | Negative |  | 4.95 | Valid |
| N13 | Negative |  | 1.02 | Negative |  | 4.58 | Valid |
| N14 | Negative |  | 0.92 | Negative |  | 4.96 | Valid |
| N15 | Negative |  | 0.99 | Negative |  | 4.97 | Valid |
| N16 | Negative |  | 1.01 | Negative |  | 4.97 | Valid |
| N17 | Negative |  | 1.32 | Negative |  | 4.95 | Valid |
| N18 | Negative |  | 0.97 | Negative |  | 5.04 | Valid |
| N19 | Negative |  | 0.97 | Negative |  | 5.02 | Valid |
| N20 | Negative |  | 0.98 | Negative |  | 5.02 | Valid |
| N21 | Negative |  | 0.96 | Negative |  | 5.00 | Valid |
| N22 | Negative |  | 1.00 | Negative |  | 4.99 | Valid |
| N23 | Negative |  | 0.99 | Negative |  | 5.02 | Valid |
| N24 | Negative |  | 0.96 | Negative |  | 5.04 | Valid |
| N25 | Negative |  | 1.03 | Negative |  | 5.04 | Valid |
| N26 | Negative |  | 1.04 | Negative |  | 5.08 | Valid |
| N27 | Negative |  | 1.02 | Negative |  | 5.54 | Valid |
| N28 | Negative |  | 1.05 | Negative |  | 5.69 | Valid |
| N29 | Negative |  | 1.06 | Negative |  | 5.70 | Valid |
| N30 | Negative |  | 1.08 | Negative |  | 5.70 | Valid |
| N31 | Negative |  | 1.08 | Negative |  | 5.75 | Valid |
| N32 | Negative |  | 1.03 | Negative |  | 5.78 | Valid |
| N33 | Negative |  | 1.02 | Negative |  | 5.77 | Valid |
| N34 | Negative |  | 0.97 | Negative |  | 5.76 | Valid |
| N35 | Negative |  | 1.00 | Negative |  | 5.74 | Valid |
| N36 | Negative |  | 1.01 | Negative |  | 5.83 | Valid |
| N37 | Negative |  | 1.01 | Negative |  | 5.75 | Valid |
| N38 | Negative |  | 1.07 | Negative |  | 5.82 | Valid |
| N39 | Negative |  | 0.99 | Negative |  | 5.72 | Valid |
| N40 | Negative |  | 0.99 | Negative |  | 5.78 | Valid |
| N41 | Negative |  | 1.02 | Negative |  | 5.75 | Valid |
| N42 | Negative |  | 1.13 | Negative |  | 5.78 | Valid |
| N43 | Negative |  | 1.12 | Negative |  | 5.82 | Valid |
| N44 | Negative |  | 1.10 | Negative |  | 5.83 | Valid |
| N45 | Negative |  | 1.09 | Negative |  | 5.78 | Valid |
| N46 | Negative |  | 1.11 | Negative |  | 5.80 | Valid |
| N47 | Negative |  | 1.05 | Negative |  | 5.86 | Valid |
| N48 | Negative |  | 1.10 | Negative |  | 5.86 | Valid |
| N49 | Negative |  | 0.84 | Negative |  | 5.83 | Valid |
| N50 | Negative |  | 1.11 | Negative |  | 5.84 | Valid |
| N51 | Negative |  | 1.09 | Negative |  | 5.83 | Valid |
| N52 | Negative |  | 0.95 | Negative |  | 5.82 | Valid |
| N53 | Negative |  | 1.09 | Negative |  | 5.78 | Valid |
| N54 | Negative |  | 1.08 | Negative |  | 5.69 | Valid |
| N55 | Negative |  | 1.06 | Negative |  | 5.72 | Valid |
| N56 | Negative |  | 1.05 | Negative |  | 5.78 | Valid |
| N57 | Negative |  | 1.08 | Negative |  | 5.80 | Valid |
| N58 | Negative |  | 1.07 | Negative |  | 5.80 | Valid |
| N59 | Negative |  | 1.05 | Negative |  | 5.24 | Valid |
| N60 | Negative |  | 0.91 | Negative |  | 5.73 | Valid |
| N61 | Negative |  | 1.04 | Negative |  | 5.25 | Valid |
| N62 | Negative |  | 1.06 | Negative |  | 5.24 | Valid |
| N63 | Negative |  | 1.02 | Negative |  | 4.71 | Valid |
| N64 | Negative |  | 1.02 | Negative |  | 4.60 | Valid |
| N65 | Negative |  | 0.93 | Negative |  | 4.94 | Valid |
| N66 | Negative |  | 1.17 | Negative |  | 4.41 | Valid |
| N67 | Negative |  | 1.15 | Negative |  | 4.69 | Valid |
| N68 | Negative |  | 1.18 | Negative |  | 4.47 | Valid |
| N69 | Negative |  | 1.19 | Negative |  | 4.60 | Valid |
| N70 | Negative |  | 1.21 | Negative |  | 4.73 | Valid |
| N71 | Negative |  | 1.20 | Negative |  | 4.75 | Valid |
| N72 | Negative |  | 1.18 | Negative |  | 4.70 | Valid |
| N73 | Negative |  | 1.18 | Negative |  | 4.66 | Valid |
| N74 | Negative |  | 1.17 | Negative |  | 4.79 | Valid |
| N75 | Negative |  | 1.15 | Negative |  | 3.60 | Valid |
| N76 | Negative |  | 1.16 | Negative |  | 3.61 | Valid |
| N77 | Negative |  | 1.17 | Negative |  | 3.60 | Valid |
| N78 | Negative |  | 1.16 | Negative |  | 3.59 | Valid |
| N79 | Negative |  | 1.13 | Negative |  | 3.50 | Valid |
| N80 | Negative |  | 1.07 | Negative |  | 3.62 | Valid |
| N81 | Negative |  | 1.15 | Negative |  | 3.63 | Valid |
| N82 | Negative |  | 1.14 | Negative |  | 3.58 | Valid |
| N83 | Negative |  | 1.11 | Negative |  | 3.57 | Valid |
| N84 | Negative |  | 1.10 | Negative |  | 3.57 | Valid |
| N85 | Negative |  | 1.12 | Negative |  | 3.60 | Valid |
| N86 | Negative |  | 1.09 | Negative |  | 3.59 | Valid |
| N87 | Negative |  | 1.06 | Negative |  | 3.63 | Valid |
| N88 | Negative |  | 1.06 | Negative |  | 3.59 | Valid |
| N89 | Negative |  | 1.06 | Negative |  | 3.61 | Valid |
| N90 | Negative |  | 1.07 | Negative |  | 3.60 | Valid |
| N91 | Negative |  | 1.06 | Negative |  | 3.56 | Valid |
| N92 | Negative |  | 1.02 | Negative |  | 3.59 | Valid |
| N93 | Negative |  | 1.06 | Negative |  | 3.54 | Valid |
| N94 | Negative |  | 1.06 | Negative |  | 3.54 | Valid |
| N95 | Negative |  | 1.05 | Negative |  | 3.55 | Valid |
| N96 | Negative |  | 1.06 | Negative |  | 3.53 | Valid |
| N97 | Negative |  | 1.04 | Negative |  | 3.61 | Valid |
| N98 | Negative |  | 0.99 | Negative |  | 3.28 | Valid |
| N99 | Negative |  | 1.00 | Negative |  | 3.09 | Valid |
| N100 | Negative |  | 0.99 | Negative |  | 3.19 | Valid |
| N101 | Negative |  | 0.93 | Negative |  | 3.25 | Valid |
| N102 | Negative |  | 0.98 | Negative |  | 3.13 | Valid |
| N103 | Negative |  | 0.95 | Negative |  | 4.36 | Valid |
| N104 | Negative |  | 0.96 | Negative |  | 4.35 | Valid |
| N105 | Negative |  | 0.96 | Negative |  | 4.80 | Valid |

N: negative sample identification number
