# Supplementary material for: Evaluation of a Lyophilized CRISPR-Cas12 Assay for a Sensitive, Specific, and Rapid Detection of SARS-CoV-2
Source: Viruses. 2021 Mar 5;13(3):420. doi: 10.3390/v13030420 (PMC7998296; doi:10.3390/v13030420)
Supplement: Supplementary file 1 [file viruses-13-00420-s001.zip › viruses-1106900-supplementary/SuppData/Table S3.docx]

**Table S3.** Lyo-CRISPR results of 105 positive RT-qPCR samples

| Sample Id |  | GeneFinder | | | | |  | | Lyo-CRISPR-SARS-CoV-2 | | | | |
| --- | --- | --- | --- | --- | --- | --- | --- | --- | --- | --- | --- | --- | --- |
|  |  | RdRp gene | N gene | E gene |  |  | | N gene | | |  | RNAseP | |
|  |  | Ct | Ct | Ct | Result |  | | R (IF_t20_/IF_t20NTC_) | | Result |  | R (IF_t20_/IF_t20NTC_) | Result |
| P1 |  | 12,43 | 11,45 | 11,36 | Positive |  | | 4,80 | | Positive |  | 5,65 | Valid |
| P2 |  | 14,63 | 11,54 | 13,36 | Positive |  | | 4,52 | | Positive |  | 5,65 | Valid |
| P3 |  | 10,83 | 14,07 | 13,14 | Positive |  | | 4,80 | | Positive |  | 5,61 | Valid |
| P4 |  | 17,93 | 16,08 | 17,33 | Positive |  | | 4,48 | | Positive |  | 5,65 | Valid |
| P5 |  | 15,89 | 16,94 | 17,22 | Positive |  | | 4,73 | | Positive |  | 5,61 | Valid |
| P6 |  | 17,91 | 18,11 | 14,97 | Positive |  | | 4,54 | | Positive |  | 5,22 | Valid |
| P7 |  | 18,97 | 18,17 | 20,32 | Positive |  | | 3,36 | | Positive |  | 5,24 | Valid |
| P8 |  | 16,78 | 18,42 | 21,02 | Positive |  | | 4,79 | | Positive |  | 5,66 | Valid |
| P9 |  | 17,37 | 18,56 | 18,06 | Positive |  | | 2,86 | | Positive |  | 3,56 | Valid |
| P10 |  | 18,65 | 18,91 | 20,84 | Positive |  | | 4,82 | | Positive |  | 5,59 | Valid |
| P11 |  | 18,19 | 19,01 | 20,01 | Positive |  | | 3,52 | | Positive |  | 5,43 | Valid |
| P12 |  | 18,76 | 19,06 | 25,58 | Positive |  | | 3,88 | | Positive |  | 3,59 | Valid |
| P13 |  | 19,89 | 19,11 | 16,55 | Positive |  | | 3,79 | | Positive |  | 5,27 | Valid |
| P14 |  | 20,42 | 19,55 | 18,62 | Positive |  | | 4,52 | | Positive |  | 5,23 | Valid |
| P15 |  | 20,61 | 19,93 | 19,83 | Positive |  | | 3,93 | | Positive |  | 5,16 | Valid |
| P16 |  | 16,91 | 20,02 | 18,68 | Positive |  | | 2,52 | | Positive |  | 3,50 | Valid |
| P17 |  | 19,69 | 20,16 | 17,11 | Positive |  | | 3,49 | | Positive |  | 5,14 | Valid |
| P18 |  | 23,40 | 20,22 | 20,00 | Positive |  | | 4,80 | | Positive |  | 5,61 | Valid |
| P19 |  | 21,42 | 20,82 | 23,25 | Positive |  | | 3,33 | | Positive |  | 5,67 | Valid |
| P20 |  | 22,05 | 21,06 | 18,78 | Positive |  | | 3,76 | | Positive |  | 4,94 | Valid |
| P21 |  | 27,71 | 21,09 | 21,36 | Positive |  | | 3,70 | | Positive |  | 5,28 | Valid |
| P22 |  | 21,05 | 21,10 | 21,50 | Positive |  | | 3,96 | | Positive |  | 5,67 | Valid |
| P23 |  | 19,12 | 21,12 | 19,08 | Positive |  | | 3,42 | | Positive |  | 3,62 | Valid |
| P24 |  | 20,15 | 21,32 | 20,18 | Positive |  | | 3,78 | | Positive |  | 5,62 | Valid |
| P25 |  | 23,16 | 21,33 | 21,08 | Positive |  | | 3,51 | | Positive |  | 5,55 | Valid |
| P26 |  | 21,90 | 21,40 | 23,39 | Positive |  | | 3,14 | | Positive |  | 4,73 | Valid |
| P27 |  | 19,52 | 21,42 | 18,89 | Positive |  | | 3,09 | | Positive |  | 3,59 | Valid |
| P28 |  | 24,01 | 21,45 | 20,15 | Positive |  | | 4,83 | | Positive |  | 5,62 | Valid |
| P29 |  | 20,31 | 21,45 | 19,50 | Positive |  | | 3,23 | | Negative |  | 5,60 | Valid |
| P30 |  | 22,98 | 21,50 | 21,59 | Positive |  | | 3,86 | | Positive |  | 3,27 | Valid |
| P31 |  | 30,17 | 21,52 | 21,94 | Positive |  | | 3,36 | | Positive |  | 3,20 | Valid |
| P32 |  | 20,79 | 21,52 | 21,32 | Positive |  | | 3,57 | | Positive |  | 5,48 | Valid |
| P33 |  | 21,38 | 21,65 | 22,75 | Positive |  | | 3,78 | | Positive |  | 5,59 | Valid |
| P34 |  | 25,84 | 21,70 | 21,40 | Positive |  | | 3,12 | | Positive |  | 5,67 | Valid |
| P35 |  | 22,76 | 21,81 | 20,59 | Positive |  | | 3,44 | | Positive |  | 5,25 | Valid |
| P36 |  | 24,21 | 22,02 | 23,45 | Positive |  | | 3,76 | | Positive |  | 5,21 | Valid |
| P37 |  | 24,47 | 22,06 | 25,20 | Positive |  | | 3,29 | | Positive |  | 5,23 | Valid |
| P38 |  | 21,65 | 22,11 | 20,11 | Positive |  | | 4,84 | | Positive |  | 5,64 | Valid |
| P39 |  | 30,78 | 22,14 | 26,19 | Positive |  | | 3,23 | | Positive |  | 3,19 | Valid |
| P40 |  | 23,39 | 22,15 | 24,01 | Positive |  | | 2,88 | | Positive |  | 5,63 | Valid |
| P41 |  | 32,32 | 22,26 | 23,48 | Positive |  | | 3,33 | | Positive |  | 5,13 | Valid |
| P42 |  | 21,72 | 22,35 | 21,78 | Positive |  | | 3,59 | | Positive |  | 5,62 | Valid |
| P43 |  | 28,86 | 22,38 | 33,80 | Positive |  | | 2,74 | | Positive |  | 5,63 | Valid |
| P44 |  | 21,66 | 22,40 | 20,30 | Positive |  | | 3,90 | | Positive |  | 3,23 | Valid |
| P45 |  | 25,19 | 22,42 | 24,19 | Positive |  | | 3,76 | | Positive |  | 5,10 | Valid |
| P46 |  | 22,77 | 22,45 | 19,96 | Positive |  | | 2,50 | | Positive |  | 3,56 | Valid |
| P47 |  | 23,37 | 22,51 | 22,91 | Positive |  | | 3,24 | | Positive |  | 5,55 | Valid |
| P48 |  | 31,61 | 22,53 | 29,68 | Positive |  | | 3,15 | | Positive |  | 5,67 | Valid |
| P49 |  | 23,94 | 22,75 | 23,80 | Positive |  | | 3,64 | | Positive |  | 3,22 | Valid |
| P50 |  | 22,17 | 22,90 | 20,40 | Positive |  | | 3,37 | | Positive |  | 3,29 | Valid |
| P51 |  | 24,37 | 23,12 | 21,87 | Positive |  | | 3,39 | | Positive |  | 5,58 | Valid |
| P52 |  | 24,64 | 23,24 | 21,75 | Positive |  | | 3,82 | | Positive |  | 5,61 | Valid |
| P53 |  | 25,05 | 23,38 | 21,55 | Positive |  | | 3,51 | | Positive |  | 5,56 | Valid |
| P54 |  | 23,23 | 23,38 | 21,65 | Positive |  | | 3,46 | | Positive |  | 4,19 | Valid |
| P55 |  | 23,46 | 23,50 | 22,90 | Positive |  | | 3,40 | | Positive |  | 3,28 | Valid |
| P56 |  | 22,45 | 23,59 | 25,25 | Positive |  | | 3,59 | | Positive |  | 2,97 | Valid |
| P57 |  | 22,05 | 23,89 | 21,69 | Positive |  | | 4,48 | | Positive |  | 5,13 | Valid |
| P58 |  | 23,63 | 23,91 | 22,47 | Positive |  | | 3,54 | | Positive |  | 5,28 | Valid |
| P59 |  | 33,14 | 23,92 | 32,17 | Positive |  | | 2,84 | | Positive |  | 3,55 | Valid |
| P60 |  | 25,41 | 24,68 | 23,44 | Positive |  | | 4,79 | | Positive |  | 5,52 | Valid |
| P61 |  | 25,01 | 24,80 | 25,04 | Positive |  | | 3,42 | | Positive |  | 5,29 | Valid |
| P62 |  | 25,10 | 24,82 | 22,29 | Positive |  | | 4,80 | | Positive |  | 5,55 | Valid |
| P63 |  | 26,80 | 24,83 | 24,29 | Positive |  | | 4,93 | | Positive |  | 5,24 | Valid |
| P64 |  | 28,14 | 25,02 | 26,13 | Positive |  | | 3,31 | | Positive |  | 4,77 | Valid |
| P65 |  | 27,76 | 25,22 | 26,99 | Positive |  | | 3,44 | | Positive |  | 5,20 | Valid |
| P66 |  | 27,91 | 25,36 | 22,44 | Positive |  | | 3,96 | | Positive |  | 3,62 | Valid |
| P67 |  | 26,57 | 25,45 | 23,36 | Positive |  | | 4,50 | | Positive |  | 5,13 | Valid |
| P68 |  | 29,36 | 25,60 | 30,50 | Positive |  | | 3,27 | | Positive |  | 5,20 | Valid |
| P69 |  | 34,09 | 25,62 | 22,48 | Positive |  | | 3,38 | | Positive |  | 5,26 | Valid |
| P70 |  | 29,06 | 26,01 | 22,22 | Positive |  | | 3,51 | | Positive |  | 5,23 | Valid |
| P71 |  | 28,48 | 26,03 | 28,38 | Positive |  | | 4,28 | | Positive |  | 5,64 | Valid |
| P72 |  | 25,19 | 26,04 | 25,75 | Positive |  | | 4,87 | | Positive |  | 5,65 | Valid |
| P73 |  | 28,66 | 26,05 | 25,38 | Positive |  | | 3,53 | | Positive |  | 5,22 | Valid |
| P74 |  | 29,44 | 26,22 | 28,44 | Positive |  | | 3,39 | | Positive |  | 5,09 | Valid |
| P75 |  | 28,90 | 26,52 | 27,71 | Positive |  | | 3,69 | | Positive |  | 5,19 | Valid |
| P76 |  | 28,91 | 26,59 | 24,31 | Positive |  | | 3,70 | | Positive |  | 5,66 | Valid |
| P77 |  | 29,01 | 26,69 | 26,56 | Positive |  | | 4,60 | | Positive |  | 5,48 | Valid |
| P78 |  | 28,71 | 27,01 | 27,18 | Positive |  | | 3,14 | | Positive |  | 5,40 | Valid |
| P79 |  | 26,41 | 27,02 | 24,67 | Positive |  | | 3,26 | | Positive |  | 3,12 | Valid |
| P80 |  | 30,92 | 27,02 | 30,07 | Positive |  | | 4,41 | | Positive |  | 5,25 | Valid |
| P81 |  | 21,00 | 27,55 | 21,09 | Positive |  | | 3,20 | | Positive |  | 5,33 | Valid |
| P82 |  | 29,99 | 27,56 | 29,64 | Positive |  | | 3,29 | | Positive |  | 4,70 | Valid |
| P83 |  | 30,07 | 28,03 | 26,36 | Positive |  | | 4,38 | | Positive |  | 5,22 | Valid |
| P84 |  | 32,53 | 28,08 | 30,68 | Positive |  | | 3,26 | | Positive |  | 5,37 | Valid |
| P85 |  | 30,05 | 28,29 | 29,51 | Positive |  | | 3,29 | | Positive |  | 5,10 | Valid |
| P86 |  | 29,16 | 29,38 | 29,40 | Positive |  | | 4,34 | | Positive |  | 5,65 | Valid |
| P87 |  | 33,97 | 29,40 | 32,15 | Positive |  | | 3,58 | | Positive |  | 4,56 | Valid |
| P88 |  | 27,28 | 29,63 | 28,63 | Positive |  | | 4,73 | | Positive |  | 5,65 | Valid |
| P89 |  | 29,99 | 30,06 | 24,98 | Positive |  | | 4,31 | | Positive |  | 5,60 | Valid |
| P90 |  | 33,03 | 30,11 | 32,75 | Positive |  | | 3,16 | | Positive |  | 4,92 | Valid |
| P91 |  | 31,95 | 30,11 | 21,92 | Positive |  | | 3,18 | | Positive |  | 5,60 | Valid |
| P92 |  | 31,80 | 30,31 | 28,29 | Positive |  | | 4,80 | | Positive |  | 5,57 | Valid |
| P93 |  | 31,80 | 30,39 | 29,29 | Positive |  | | 4,78 | | Positive |  | 5,56 | Valid |
| P94 |  | 30,11 | 30,53 | 32,22 | Positive |  | | 3,59 | | Positive |  | 4,67 | Valid |
| P95 |  | 34,26 | 30,56 | 31,04 | Positive |  | | 4,62 | | Positive |  | 4,54 | Valid |
| P96 |  | 32,68 | 30,64 | 30,81 | Positive |  | | 3,14 | | Positive |  | 4,74 | Valid |
| P97 |  | 30,54 | 31,36 | 30,24 | Positive |  | | 4,54 | | Positive |  | 4,79 | Valid |
| P98 |  | 33,82 | 31,94 | 31,25 | Positive |  | | 3,24 | | Positive |  | 4,82 | Valid |
| P99 |  | 33,68 | 32,05 | 29,89 | Positive |  | | 4,87 | | Positive |  | 4,54 | Valid |
| P100 |  | 35,57 | 32,25 | 34,25 | Positive |  | | 3,18 | | Positive |  | 4,71 | Valid |
| P101 |  | 34,35 | 32,52 | 30,69 | Positive |  | | 3,32 | | Positive |  | 4,86 | Valid |
| P102 |  | 32,23 | 32,80 | 33,31 | Positive |  | | 4,74 | | Positive |  | 5,53 | Valid |
| P103 |  | 40,02 | 33,45 | 30,61 | Positive |  | | 3,44 | | Positive |  | 4,83 | Valid |
| P104 |  | 34,23 | 33,63 | 21,25 | Positive |  | | 3,22 | | Positive |  | 5,66 | Valid |
| P105 |  | 35,25 | 36,90 | 34,27 | Positive |  | | 4,80 | | Positive |  | 5,66 | Valid |

P: positive sample identification number; Ct: Cycle threshold.
