# Supplementary material for: Evaluation of a Lyophilized CRISPR-Cas12 Assay for a Sensitive, Specific, and Rapid Detection of SARS-CoV-2
Source: Viruses. 2021 Mar 5;13(3):420. doi: 10.3390/v13030420 (PMC7998296; doi:10.3390/v13030420)
Supplement: Supplementary file 1 [file viruses-13-00420-s001.zip › viruses-1106900-supplementary/SuppData/Table S4.docx]

**Table S4.** Lyo-CRISPR results of 30 samples collected in lysis buffer

| Sample Id |  | GeneFinder RT-qPCR | | | |  | Lyo-CRISPR SARS-CoV-2 | | | | |
| --- | --- | --- | --- | --- | --- | --- | --- | --- | --- | --- | --- |
|  |  | RdRp gene | N gene | E gene |  |  | N gene | |  | RNAseP | |
|  |  | Ct | Ct | Ct | Result |  | R (IF_t20_/IF_t20NTC_) | Result |  | R (IF_t20_/IF_t20NTC_) | Result |
| PLB1 |  | 9,10 | 9,17 | 8,30 | Positive |  | 3,03 | Positive |  | 3,40 | Valid |
| PLB2 |  | 10,86 | 11,61 | 9,61 | Positive |  | 3,34 | Positive |  | 3,36 | Valid |
| PLB3 |  | 13,40 | 12,77 | 11,51 | Positive |  | 3,87 | Positive |  | 3,40 | Valid |
| PLB4 |  | 16,88 | 16,68 | 15,40 | Positive |  | 3,56 | Positive |  | 3,45 | Valid |
| PLB5 |  | 17,97 | 17,77 | 17,34 | Positive |  | 3,79 | Positive |  | 3,41 | Valid |
| PLB6 |  | 17,06 | 18,28 | 19,49 | Positive |  | 3,80 | Positive |  | 3,41 | Valid |
| PLB7 |  | 19,06 | 19,88 | 18,88 | Positive |  | 3,68 | Positive |  | 3,74 | Valid |
| PLB8 |  | 21,81 | 20,89 | 21,63 | Positive |  | 3,81 | Positive |  | 3,40 | Valid |
| PLB9 |  | 19,65 | 20,91 | 18,13 | Positive |  | 3,05 | Positive |  | 3,70 | Valid |
| PLB10 |  | 20,01 | 21,68 | 19,77 | Positive |  | 3,20 | Positive |  | 3,68 | Valid |
| PLB11 |  | 22,70 | 22,62 | 21,76 | Positive |  | 3,79 | Positive |  | 3,40 | Valid |
| PLB12 |  | 21,55 | 22,92 | 22,67 | Positive |  | 3,75 | Positive |  | 3,83 | Valid |
| PLB13 |  | 24,01 | 24,09 | 25,16 | Positive |  | 2,80 | Positive |  | 3,41 | Valid |
| PLB14 |  | 22,01 | 25,02 | 21,08 | Positive |  | 3,77 | Positive |  | 3,84 | Valid |
| PLB15 |  | 17,67 | 26,55 | 24,21 | Positive |  | 3,72 | Positive |  | 3,40 | Valid |
| PLB16 |  | 24,51 | 26,91 | 24,63 | Positive |  | 3,48 | Positive |  | 3,51 | Valid |
| PLB17 |  | 25,73 | 27,39 | 26,71 | Positive |  | 3,05 | Positive |  | 3,59 | Valid |
| PLB18 |  | 26,98 | 29,61 | 16,41 | Positive |  | 3,85 | Positive |  | 3,40 | Valid |
| PLB19 |  | 31,74 | 30,61 | 3,43 | Positive |  | 3,75 | Positive |  | 3,36 | Valid |
| PLB20 |  | 29,71 | 32,08 | 30,47 | Positive |  | 3,31 | Positive |  | 3,51 | Valid |
| PLB21 |  | 35,60 | 36,65 | 34,32 | Positive |  | 1,07 | Negative |  | 3,54 | Valid |
| NLB1 |  | - | - | - | Negative |  | 1,18 | Negative |  | 4,15 | Valid |
| NLB2 |  | - | - | - | Negative |  | 1,10 | Negative |  | 3,99 | Valid |
| NLB3 |  | - | - | - | Negative |  | 1,19 | Negative |  | 3,77 | Valid |
| NLB4 |  | - | - | - | Negative |  | 1,15 | Negative |  | 3,81 | Valid |
| NLB5 |  | - | - | - | Negative |  | 1,14 | Negative |  | 3,80 | Valid |
| NLB6 |  | - | - | - | Negative |  | 1,03 | Negative |  | 3,87 | Valid |
| NLB7 |  | - | - | - | Negative |  | 1,03 | Negative |  | 3,82 | Valid |
| NLB8 |  | - | - | - | Negative |  | 1,10 | Negative |  | 3,41 | Valid |
| NLB9 |  | - | - | - | Negative |  | 1,05 | Negative |  | 3,69 | Valid |

PLB: positive sample in lysis buffer; NLB: negative sample in lysis buffer; Ct: Cycle threshold.
